# Indications of a ferromagnetic quantum critical point in $\textrm{SmN}_{1-\delta }$

**DOI:** 10.1038/s41598-023-46911-5

**Published:** 2023-11-13

**Authors:** W. F. Holmes-Hewett, K. Van Koughnet, J. D. Miller, E. X. M. Trewick, B. J. Ruck, H. J. Trodahl, R. G. Buckley

**Affiliations:** 1 Robinson Research Institute, Victoria University of Wellington, P.O. Box 33436, Petone, 5046 New Zealand; 2 School of Chemical and Physical Sciences, Victoria University of Wellington, P.O. Box 600, Wellington, 6140 New Zealand; 3 MacDiarmid Institute for Advanced Materials and Nanotechnology, P.O. Box 600, Wellington, 6140 New Zealand

**Keywords:** Electronic properties and materials, Magnetic properties and materials, Phase transitions and critical phenomena, Semiconductors, Superconducting properties and materials

## Abstract

We investigate the previously observed superconductivity in ferromagnetic SmN in the context of the breakdown of order between two magnetic phases. Nitrogen vacancy doped SmN$_{1-\delta }$ is a semiconductor which lies in the intermediary between ferromagnetic SmN and anti-ferromagnetic Sm. Optical data reported here corroborate the prediction that electrical transport is mediated by Sm 4*f* defect states, and electrical transport measurements characterise the metal-insulator transition over the doping range. Our measurements show that the superconducting state in nitrogen vacancy doped $\textrm{SmN}_{1-\delta }$ is the most robust near the breakdown of magnetic order, and indicate the location of a quantum critical point. Furthermore we provide additional evidence that the superconducting state is formed from majority spin electrons and thus of unconventional S = 1 type.

## Introduction

The borders of ordered magnetic states in *f*-electron systems are a common source of emergent phenomena, including unconventional superconductivity and heavy fermion behaviour^1–6^. Significant effort has been expended understanding these phenomena in terms of zero-temperature phase transitions and their associated quantum critical points (QCP), which are commonly found near spin-order instabilities presenting as zero-temperature magnetic transitions^7–12^. Traditionally QCPs lie at boundaries between antiferromagnetic (AFM) and paramagnetic (PM) phases. However there is recent interest in ferromagnetic (FM) phase boundaries^13–17^. Lanthanide compounds have led as examples, and although the rare earth mononitrides (*Ln*N, *Ln* a lanthanide) were proposed already some years ago as likely heavy-fermion candidates^18–20^, that prediction has not yet been explored in great detail.

The *Ln*N (*Ln*$^{3+}$N$^{3-}$) are poised at a metal-insulator boundary, and ongoing studies over the last half century have placed them variously on the two sides of that boundary^21–23^. Recent thin films are mostly on the insulator side, showing signatures of a dopable semiconducting ground state with a narrow but clear band gap^24–27^. Electron doping in thin films can be routinely controlled by a residual concentration V$_N$ of nitrogen vacancies^28–31^. The *Ln*N crystallise in the NaCl structure with lattice constants varying from 0.51 to 0.48 nm across the series^32^. Within that structure the cations form a close-packed FCC network that differs only in the stacking sequence from that in the hexagonal structures of the pure lanthanide metals. Remarkably, in addition to their similar local close-packed arrangements, the *Ln*-*Ln* separation is only slightly different; in particular the Sm-Sm separation in SmN is 0.3560 nm vs 0.3606 nm in metallic Sm, a contrast of only 1.3%.

The picture that emerges is then of a close-packed lanthanide lattice with nitrogen ions entering the network with minimal influence on the *Ln* packing density. The nitrogen ions each remove three electrons from the *Ln* 5*d*, 4*f* and 6*s* states that form the conduction channel, finally reducing the mobile electron concentration to zero in the stoichiometric *Ln*N. In the pure metallic *Ln* phase the magnetic exchange, ferromagnetic for most, is dominated by an RKKY interaction via those mobile electrons, and nesting across portions of the Fermi surface then leads to the rich range of spiral spin alignments revealed by neutron scattering studies fifty years ago^33^. In contrast the nitrides, also mostly ferromagnetic, involve an indirect exchange via the *Ln* 5*d* and N 2*p* states^34,35^.

Interest in the *Ln*N series has thrived for well over half a century, although a focus on their magnetic properties has been only rarely extended to discussions of their strong correlation^18–20^. Most attention has been expended on GdN, with the series’ highest Curie temperature of $\sim$ 65 K. In the half-filled 4*f* level, the $^8$S$_{7/2}$ configuration dictates that there is no orbital contribution to the magnetic moment; it is an entirely conventional spin-only ferromagnetic compound. Furthermore the wide spin-splitting places the 4*f* bands far from the Fermi energy such that they do not contribute to electron transport. Electron doping in GdN is usually facilitated by nitrogen vacancies which lift the Fermi energy into the dispersive *Ln* 5*d* bands^28^.

The lighter members of the *Ln*N feature majority spin 4*f* bands lying within the conduction band (CB) precipitating dopable strongly correlated 4*f* states^36–38^. The Sm$^{3+}$ ion in SmN has five 4*f* electrons, ensuring that there are two empty majority-spin 4*f* bands threading the 5*d* CB^39–41^. The inter-ion exchange precipitates ferromagnetic spin alignment of those five occupied 4*f* states below $\sim$ 30 K. However, within the $^6$H$_{5/2}$ state the spin magnetic moment is opposed by an orbital moment of similar magnitude so that the *net* magnetic moment is nearly zero; SmN displays ferromagnetic alignment and exchange split bands but has a near zero net moment of $\sim$ 0.035 $\mu _B$ per Sm ion^42–44^.

When nitrogen vacancies are induced in the crystal the two unfilled majority spin 4*f* states on the six Sm ions which coordinate the vacancy are drawn into the intrinsic band gap, and towards the valance band maximum with increasing doping levels^45^. The three electrons released by a nitrogen vacancy thus do not appear in the intrinsic Sm 5*d* conduction band minimum (CBM), they rather occupy the majority spin 4*f* states on those now mixed Sm$^{3+}$/Sm$^{2+}$ ions surrounding the vacancy. These states appear $\sim$ 1 eV lower than those on fully nitrogen coordinated ions, pinning the Fermi energy to the mid-gap region, where they hybridise with both the 5*d* and N 2*p*^45^.

Within the *Ln*N series the Sm/SmN pair stands out as the one in which the end points have contrasting magnetic order, ferromagnetic below 30 K in the mononitride but antiferromagnetic below 100 K in metallic Sm^46,47^. The situation is reminiscent of those materials where competing magnetic ground states are traversed via doping^48^, materials which display emergent correlated behaviours in the intermediary between these ground states. Significantly, SmN is also the only *Ln*N in which superconductivity has been reported across the doping range range. This has been observed in heavily doped (i.e. nitrogen deficient) SmN$_{1-\delta }$^49^, motivating a search for heavy-fermion superconductivity that is commonly found near a QCP^2,50,51^. Here we report a combined experimental and computational study of the band structure and defect states that precipitate superconductivity in SmN$_{1-\delta }$ and measurements which indicate the location of a quantum critical point.

**Figure 1:**
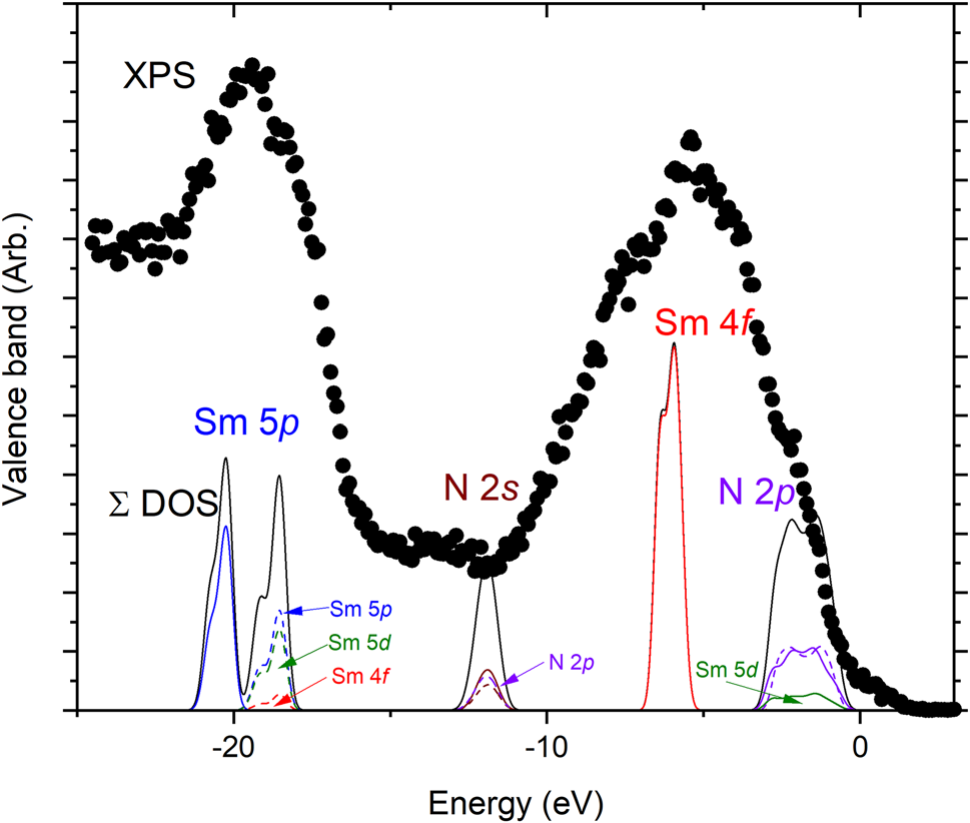
XPS measurements (black circles) and DOS calculations (lines) for SmN. The total DOS (majority and minority spin) are shown in solid black lines, the contribution from separate orbitals are shown in various colours (majority spin-solid lines, minority spin-dashed lines), for clarity only the significant contributions to each peak are shown.

**Figure 2:**
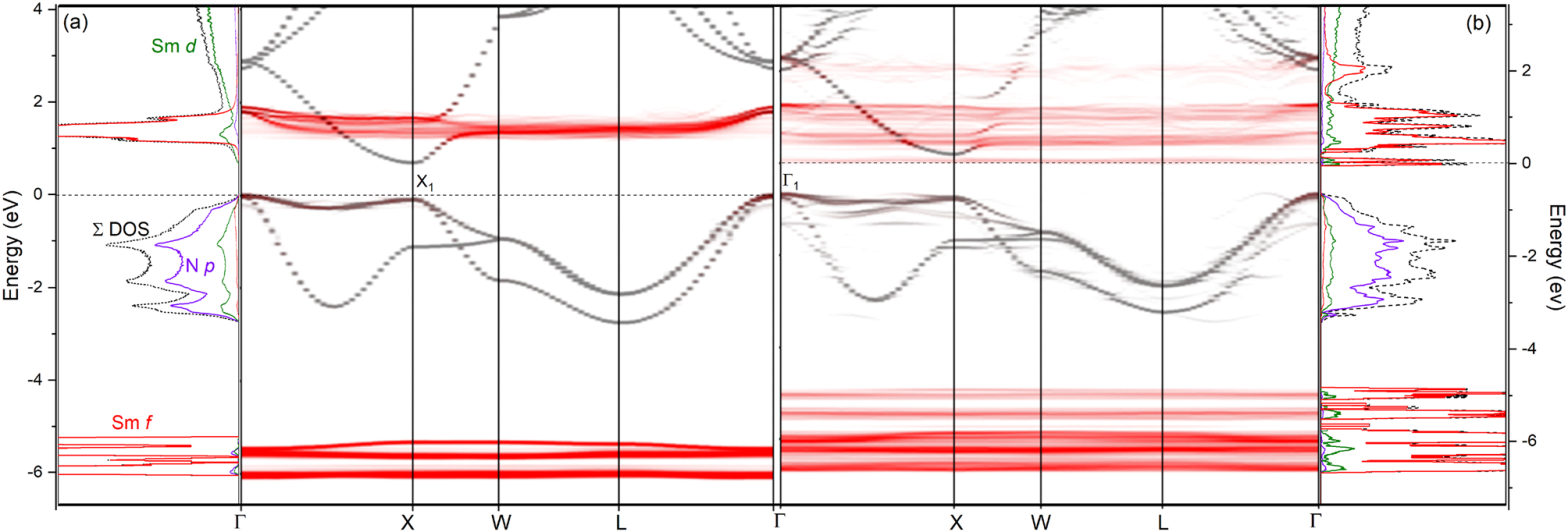
Calculated band structure and DOS for undoped (**a**) and nitrogen vacancy doped (**b**) SmN supercells. The 4*f* character of the bands in indicated in red. $\Gamma _1$ and X$_1$ indicate optical transitions present in Fig. 4.

The results are presented as follows, to being we discuss in section “Band structure” the calculated and experimental valence bands of SmN. We then discuss the calculated band structure of stochometric and nitrogen vacancy doped SmN, before moving to the experimental results in section “Electrical transport and optical spectroscopy”. The optical spectroscopy represents transitions between filled and unfilled states in the band structure, which can be used to validate the calculations of section “Band structure”, while the electrical transport measurements relate to the properties of the material in the vicinity of the Fermi energy. Finally in section “Preliminary phase diagram” we bring together the computational band structure and experimental results to build a preliminary phase diagram for SmN, and comment on the nature of the superconductivity. Full details of the experimental and computational methods can be found in section “Methods”.

## Results

### Band structure

Figure 1 shows the experimental X-ray photo-emission spectroscopy (XPS) spectrum along with the calculated density of states (DOS) in the valence band of SmN. The lowest energy feature in the experimental data, with a centre near − 19.5 eV, matches well with the calculated position of the the Sm 5*p* states. The calculation shows two spin-split peaks, while the experiment is carried out at ambient temperature in the paramagnetic phase, so the majority and minority Sm 5*p* states are degenerate at intermediate energy. Moving to higher energy the N 2*s* states are placed by the calculation at $\sim -12$ eV, however these are not clear in the measurement. One notes that the N 2*s* feature is substantially weaker in comparison with the Sm-associated features. Possibly that results from a level of resonance in the Sm features associated with similar energies between the excitation X-ray and the Sm M edge.

The valence band maximum (VBM) is formed from N 2*p* states, which the calculation finds hybridised with the majority spin Sm 5*d* states. Finally the peak in the XPS data at $\sim ~-$ 5.25 eV matches well with the calculated location of the Sm 4*f* states, of which majority spin only are present. The correspondence between the calculated Sm 4*f* states and the experimental XPS spectrum is significant as this indicates the calculated value of $U_f=6.77$ eV is appropriate (see section “Density functional theory calculations”). The XPS data are much broader than the calculated DOS, particularly for the Sm 4*f* states, as is also observed in ErN^52^. This signals the multiplet structure of the 4*f* states are not captured by our calculation. A recent series of calculations using a density-functional + dynamical mean-field theory approach can be found in reference^53^, where these effects are more accurately captured in the stochometric *Ln*N and in EuN^54^.

Typically the band-structure of undoped SmN is based on the two-atom primitive unit cell, which lacks any disorder. A physical crystal will deviate somewhat from the pristine structure imposed by these periodic boundary conditions. To investigate something closer to the physical material we have relaxed an undoped 54 atom super-cell which was seeded with a small amount of disorder. The resulting band structure, unfolded to represent the familiar bands of the primitive unit cell, is shown in Fig. 2a (for clarity the majority spin bands only are shown). The band structure shown in Fig. 2a is indeed very similar to that of the commonly reported primitive unit cell^39,45^ with the main contrast at the VBM. In the primitive unit cell calculation the VBM is at $\Gamma$ with a close to 0.5 eV drop towards the X point. In our disordered super-cell we see the VBM at $\Gamma$ is suppressed resulting in a near direct gap at the X point $(\epsilon (\Gamma )-\epsilon (\textrm{X})\sim 0.05~eV)$.

Figure 2b shows the unfolded bands of a SmN super-cell doped with a nitrogen vacancy at $\sim$ 3 % concentration. As described in our earlier work^45^, the structural disorder resulting from the vacancy results in long range periodicity and thus new defect states in the crystal. These manifest as the *ghost-like* bands, the shading of which signifies the weight of the unfolded state for a given *k*-point. The most striking feature is that the Fermi energy resides in the intrinsic gap region rather than the VBM or the CBM. It is pinned here by defect states which are largely localised to the six Sm ions which coordinate the vacancy site^45^. This is a clear contrast to the other V$_N$ doped *Ln*N studied computationally, the more simple GdN^28^ and LuN^31^, where V$_N$ doping lifts the Fermi energy into the *Ln* *5*d CB in a more conventional donor doping manner.

### Electrical transport and optical spectroscopy

**Figure 3:**
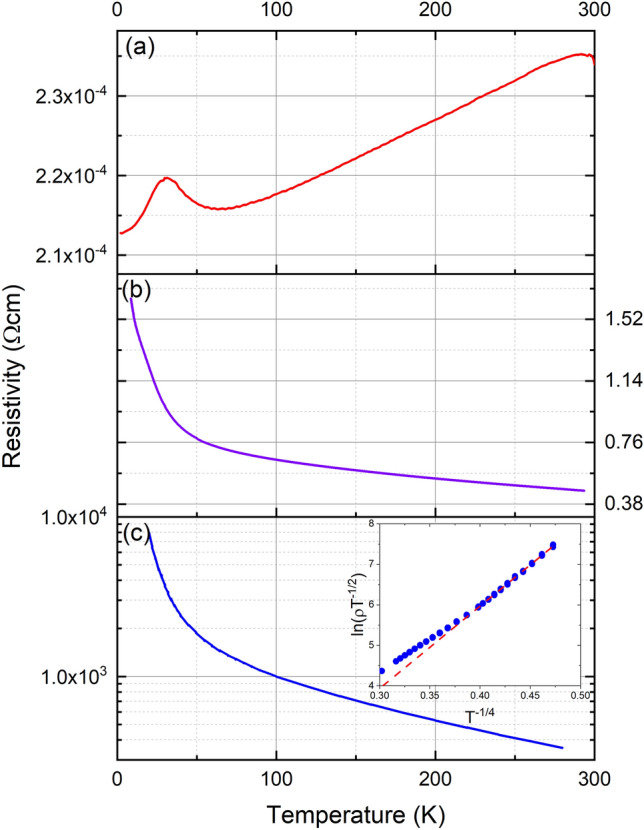
Resistivity as a function of temperature for three SmN films produced to harbour significantly different concentrations of nitrogen vacancies. Panel (**a**) shows a film with a metallic like conductivity. Panels (**b**) and (**c**) show films with non-metallic conductivity. The inset to panel (**c**) shows the data plotted appropriately to display the variable range hopping type conductivity at low temperatures.

With the computational band structure in mind we now turn to the electrical transport and optical spectroscopy measurements. The resistivity as a function of temperature in Fig. 3 show clearly the contrasting behaviour between films grown with various concentrations of V$_N$. The most conductive film in panel (a) shows a positive coefficient of temperature; it is clearly doped to degeneracy, and as such is beyond interpretation in the context of Figs. 1 and 2. The more nearly stoichiometric films show resistivities that diverge at the lowest temperatures. The most nearly stoichiometric film has a resistivity which increases strongly with decreasing temperature. The inset of panel (c) shows the measurements plotted below 120 K, with the linear dependence $\ln (\rho T^{-1/2}) \propto T^{-1/4}$ below $\sim$ 40 K characteristic of variable range hopping ^55^. The anomaly near 20 K in the conductive film is close to the ferromagnetic transition. This is a result of magnetic disorder scattering^56–58^ peaking very near the Curie temperature. A similar feature is present in our moderately doped films (not shown) and results from a band gap reduction across the Curie temperature^24^. This feature is obscured in the most resistive films by the rise in resistivity at low temperature.

**Figure 4:**
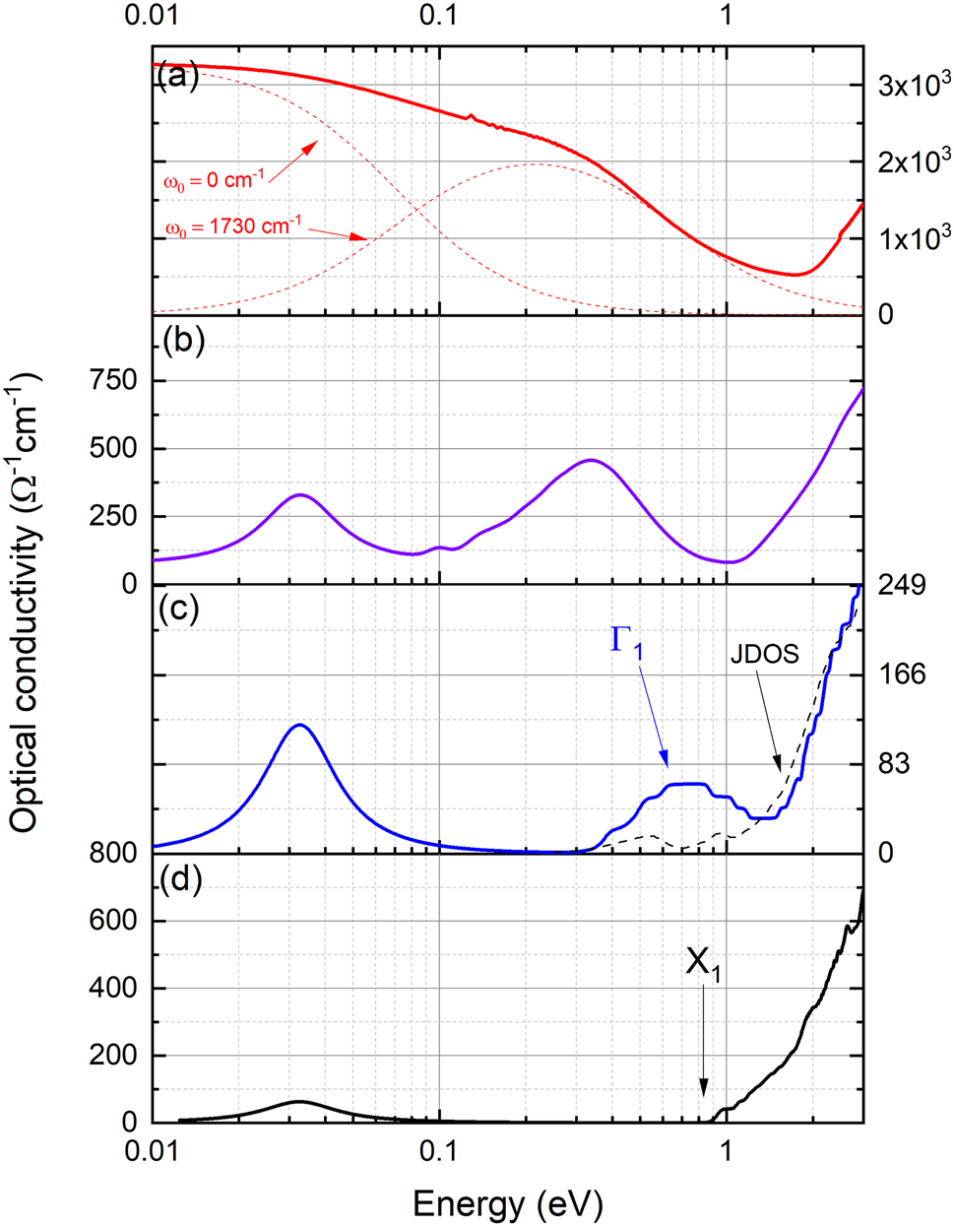
Optical conductivity for four SmN films based on reflection and transmission measurements. Panel (**c**) shows in addition the joint density of states corresponding to the band structure of Fig. 2b. The points X$_1$ and $\Gamma _1$ corrospond to optical transitions indicated in Fig. 2. The colours are consistent between Figs. 3 and 4 and represent measurements on the same films.

Far IR to near UV optical spectroscopy was performed on a series of films doped variously with nitrogen vacancies leading to dc (zero-frequency) conductivities ranging from $\sim$ 1$\times$10$^{-4}~\Omega ^{-1}$cm$^{-1}$ to $\sim$ 5000 $\Omega ^{-1}$cm$^{-1}$. Figure 4 shows the resulting optical responses of four films, two from the extremes of our conductivity and two representative of the centre. The most insulating film in panel (d) of Fig. 4 is the closest to stoichiometric that we have grown, and as such has a dc resistivity above our measurement limits and correspondingly the optical conductivity falls below our measurement limits at low energy. At $\sim$ 30 meV there is an absorption relating to the IR active TO $\Gamma$ mode vibration which we see in all *Ln*N films^59^. There is then no absorption in the MIR region before an increase near $\sim$ 1 eV signalling the onset of optical transitions. The onset of absorption is marked as X$_1$ in Fig. 4d and the corresponding optical transition is indicated in Fig. 2a. Moving to the next most insulating film in panel (c) we now see a feature begin to develop below the intrinsic optical gap, noted as $\Gamma _1$ in Fig. 4c and the corresponding optical transition in Fig. 2b. Panel (c) also shows the joint density of states (JDOS—dashed line) corresponding to the electronic structure of the material illustrated in Fig. 2b. The JDOS shows a double peaked MIR feature, the centre of which corresponds remarkably well to the peak in the MIR data. Recalling that the localised V$_N$ state spans the full range of wave vector space it is clear that the transition here involves the defect states at the Fermi energy and the relatively flat $\Gamma -$X valence band. As usual the relative strengths in the conductivity features are not fully represented by the JDOS; the coupling between the initial and final states depends critically on the dipole matrix elements that vary widely among transitions.

The moderately conductive film in panel (b) has a finite zero-frequency conductivity consistent with the electrical measurements in Fig. 3. The MIR feature has grown in magnitude and shifted to lower energies. Finally the most conductive film in panel (a) has a strong free carrier absorption at the lowest energies, which matches well the measured dc conductivity. The MIR feature has now softened with a centre near 0.2 eV. The contributions with central frequencies at $\omega _0=0$ cm$^{-1}$ and $\omega _0=1730$ cm$^{-1}$ which dominate the optical conductivity are plotted separately for clarity.

**Figure 5:**
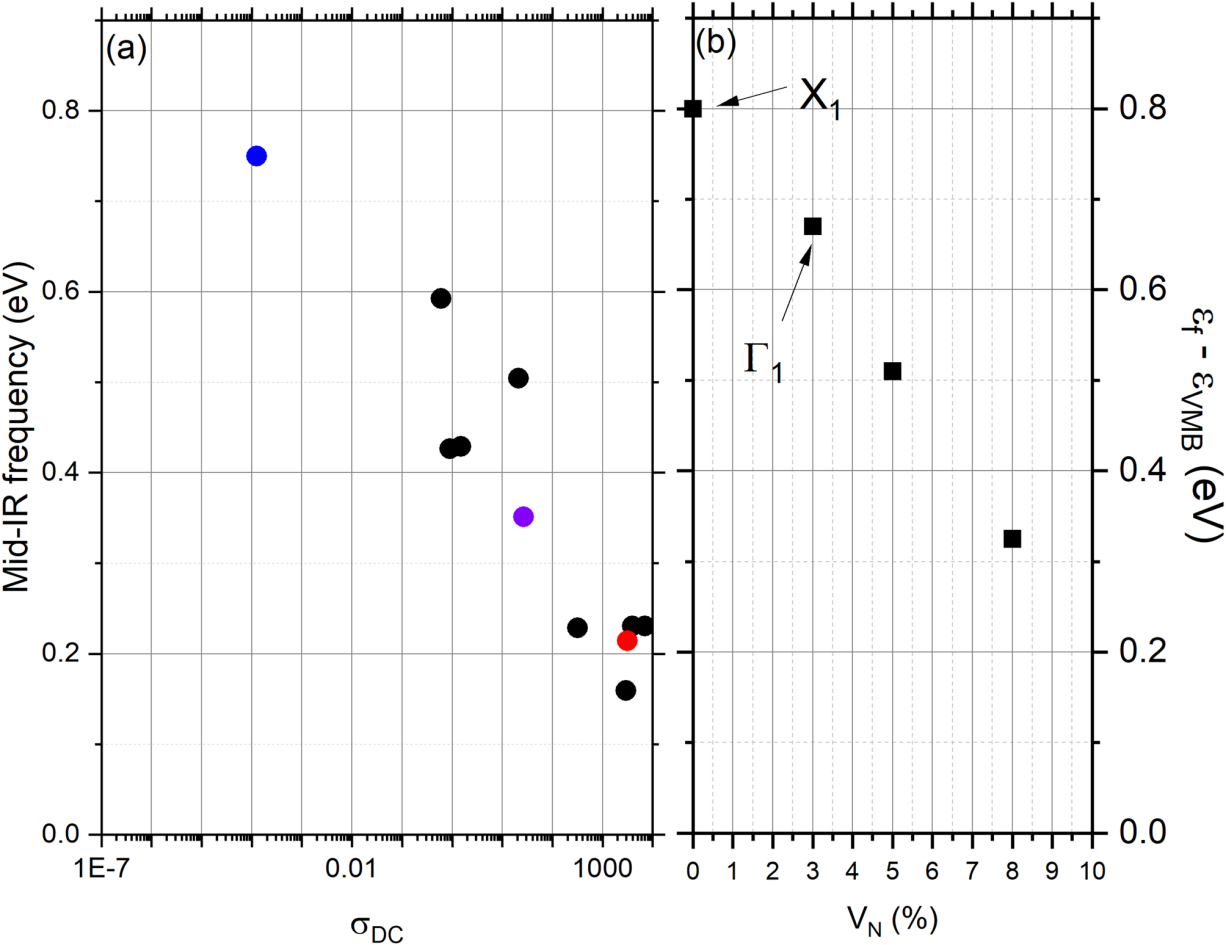
Panel (**a**) shows the centre of the mid infra-red feature for a series of SmN films. The coloured points indicated films in Figs. 3 and 4. Panel (**b**) shows the difference between the Fermi energy and valence band maximum for calculations over a range of nitrogen vacancy doping concentrations. The transitions X$_1$ and $\Gamma _1$ indicated in Figs. 2 and 4 and also labelled here.

The development of the MIR absorption is the most important change with V$_N$ across Fig. 4a–d, along with its softening to lower energy as the conductivity increases. Lorenztian fits to the feature in the full set of films yield the central energy plotted versus the conductivity in Fig. 5a. This shows a sharply reduced energy as the conductivity rises, falling from $\sim$ 0.75 eV in near-stoichiometric film to $\sim$ 0.2 eV in heavily doped films. In order to understand the softening of the MIR feature, we return to the calculated band structure in nitrogen deficient SmN^45^ and Fig. 2b in the present manuscript. As noted above a defect band dominated by the 4*f* states on the Sm ions neighbouring a vacancy forms within the SmN fundamental gap when the crystals are doped with V$_N$. The Fermi level is then pinned to these in-gap defect states, forming an increasingly extended-state band as the V$_N$ concentration, and thus doping, increases.

The present measurements (shown in Fig. 5a) indicate that the in-gap states move deeper with an increasing V$_N$ effectively pulling the Fermi energy towards the valence band maximum. As these states span the Fermi energy they can harbour both final and initial states for an optical transition involving any of the extended-state bands in the CB and VB. On that basis we look at transitions involving the mid-gap 4*f* states. These transitions effectively represent a measurement of the separation between the VBM at $\Gamma$ and the Fermi energy on one side, and the separation between the Fermi energy and the unoccupied states at the CBM on the other side. In Fig. 5b we have plotted the separation in energy between the valence band maximum and Fermi energy for the four concentrations examined computationally (note that for the 0 % doping case the value represents the minimum direct optical gap between the VB and CBM at X). The optical transitions $\Gamma _1$ and X$_1$ indicated in Figs. 2 and 4 are again indicated in Fig. 5. The agreement between the optical data and the calculation are striking and suggest that the transitions from the VBM to the defect states at the Fermi energy are the dominant contribution to the MIR feature in the optical spectra. This clarifies our previous report^60^, the MIR feature clearly tracks the defect states which are accommodated by Sm ions adjacent to the vacancy site, rather than an *intrinsic* unfilled majority spin 4*f* states.

**Figure 6:**
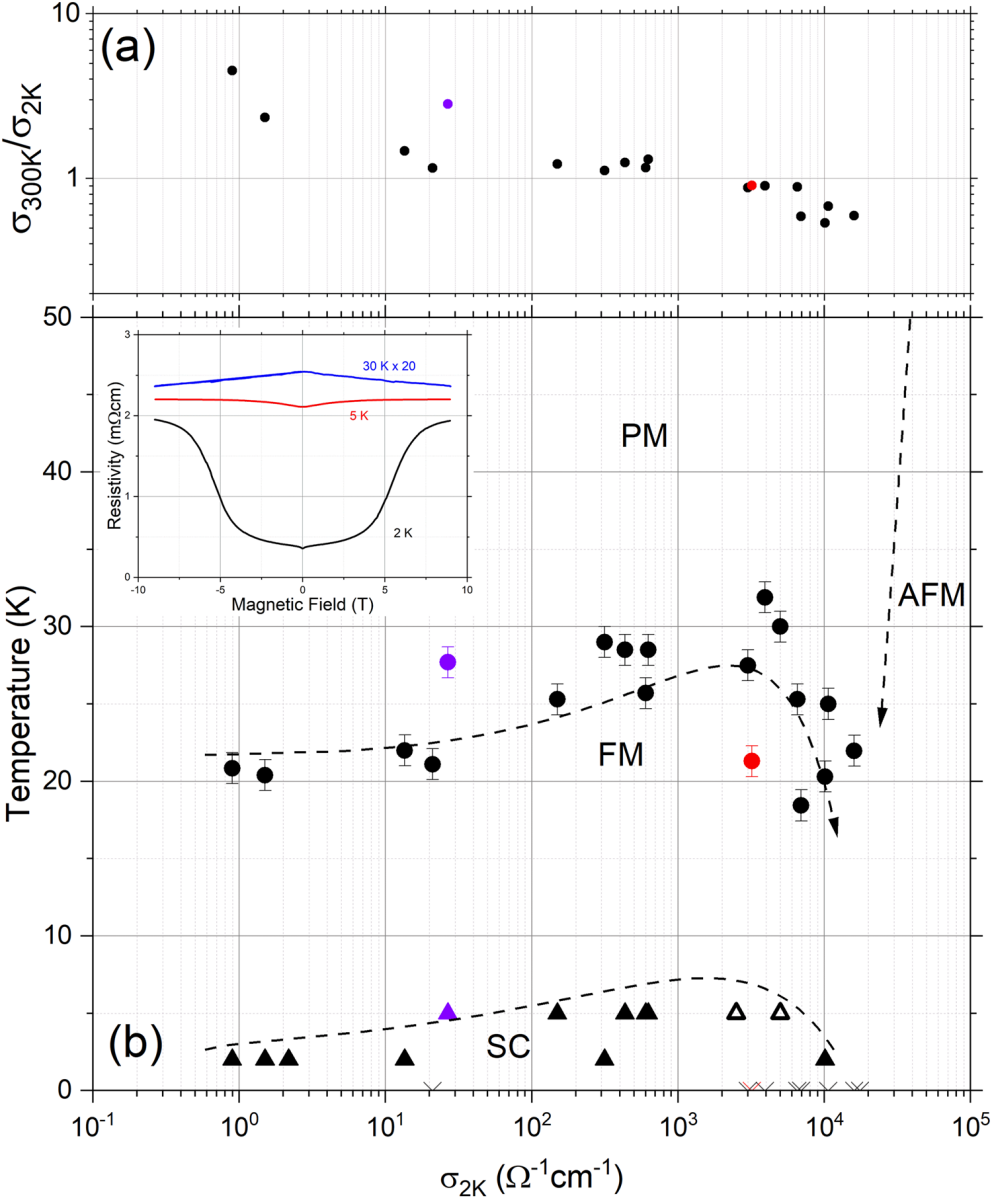
Panel (**a**) shows the ratio of 300 K conductivity to 2 K conductivity for the series of SmN films. Panel (**b**) shows a phase diagram for the series of SmN films with the paramagnetic, ferromagnetic (filled circles) and superconducting (filled triangles) phases of SmN and anti-ferromagnetic phase of metallic Sm. The two open triangles show the robust superconducting transitions already reported in reference^49^. The inset shows an example of the magneto-resistance measurements used to characterise the onset of superconductivity.

Now guided by the band structure calculations, the location of defect states lying at the Fermi energy necessarily imply that they are involved in all aspects of electron transport. One now expects that electron transport in SmN should be dominated by 4*f* in-gap states rather than extended states in the intrinsic 5*d* CB. In that regard there already exist anomalous Hall effect data that suggest 4*f* conduction in SmN^36^, rather than the 5*d* conduction seen in GdN^61^. In the dilute doping case the occupied states are localised, resulting in the variable range hopping type conductivity of Fig. 3c. As V$_N$ increases a percolation limit is found, resulting in a lower resistivity and metallic like temperature dependence, seen in the heavily doped film in Fig. 3a and the conventional Drude roll-off in the same film, shown in Fig. 4a. The transition between non-metallic and metallic conductivity can be seen in Fig. 6a which shows the ratio of 300 K conductivity to 2 K conductivity for the series of SmN films. It is significant that the SmN films which have exhibited robust superconductivity are found near this transition, which we discuss in more detail below.

### Preliminary phase diagram

Figure 6b shows a preliminary phase diagram depicting the paramagnetic, ferromagnetic and superconducting phases of SmN$_{1-\delta }$ and the anti-ferromagnetic phase of Sm metal. The order parameter of choice is carrier concentration, for which we use the low temperature conductivity as a proxy. The filled circles on the plot show the transition between the paramagnetic and ferromagnetic phases. The temperature of this transition was estimated from the peak in the resistivity as discussed previously. The Curie temperature grows with increasing conductivity from $\sim$ 20 K in the most undoped films to a maximum of $\sim$ 30 K. This suggests a carrier enhanced exchange interaction. Exactly such an enhancement is seen also in GdN^29,34^ and DyN^62^. Unlike those examples the end state of Sm metal is AFM and indeed the Curie temperature in Fig. 6b drops and finally looks to terminate near a nitrogen vacancy doping corresponding to a conductivity of $\sim$ 20,000 $\Omega ^{-1}$cm$^{-1}$.

Although we have only observed clear superconductivity (via both a Meniser effect and a zero-resistance) in a few films^49^ we regularly see the onset of a low temperature phase characterised by a resistance drop, which is often more clearly observed in the low temperature magneto-resistance. The low temperature magneto-resistance for a film is plotted in the inset to Fig. 6b. This shows the change in sign from the negative magneto-resistance common in our films in the ferromagnetic phase near 30 K to positive at 5 K and below. This is similar to the magneto-resistance in ref^49^, which we interpret as the onset of the superconducting phase. The onset of this phase is plotted as triangles in Fig. 6b. It is significant that the onset of the low temperature phase roughly tracks the onset of ferromagnetism, which is enhanced in the more conductive films before a sharp drop. The crosses on the *x*-axis of Fig. 6 show films in which we did not observe a low temperature transition or onset above our minimum temperature of 1.9 K. It is interesting that almost all films in the conductivity range of 1–1000 $\Omega ^{-1}$cm$^{-1}$ show a low temperature transition, while most films above 1000 $\Omega ^{-1}$cm$^{-1}$ do not.

The scatter in the data, particularly at high conductivity, highlight that $\sigma _{\textrm{2K}}$ is a rough proxy for the order parameter. We propose the carrier concentration, driven by nitrogen vacancy doping, is the most natural choice of order parameter and although the conductivity is proportional to this, there are other contributions, for example the scattering time, which may cause problems with this proxy, particularly in the most conductive films.

The competition between the AFM and FM phases in the region above 1000 $\Omega ^{-1}$cm$^{-1}$ is reminiscent of the competition between the Kondo and RKKY interactions proposed decades ago^63^. Within that picture the ground state was RKKY-mediated AFM, changing to PM when the Kondo interaction dominates. In the present system Fig. 6b shows a falling Curie temperature above 1000 $\Omega ^{-1}$cm$^{-1}$; the FM exchange coupling clearly weakens with the increasing dominance of the AFM interaction of Sm metal. It is significant that the superconducting transitions appears strongest just where the fall in Curie temperature shows the influence of FM/AFM competition. This is the region where a weakening inter-ion pairing mechanism would be expected to result in the strong quantum fluctuations which result in quantum critical behaviours. The superconducting transition appears precisely where one would expect within the quantum critical point scenario. This study suggests a quantum critical point in the SmN-Sm phase diagram near a nitrogen vacancy doping corresponding to 20000 $\Omega ^{-1}$cm$^{-1}$, at the breakdown of magnetic order.

The present results, along with previous experimental reports^36^, support electron transport in a completely spin-polarised defect band. Any electron pair formed in the defect band of SmN must thus have S=1 which requires an odd orbital wave function. This suggests the origins of the superconducting state may indeed not be of the conventional *s*-wave phonon mediated pairing. The more exotic spin-spin pairing mechanism of interest here is thought to be viable only near a critical point where long range magnetic order is suppressed^2,50^.

## Conclusions

We have undertaken experimental and computational studies on SmN films over a wide range of conductivities. The data show SmN exhibits a hopping type conductivity when nearly stoichiometric, and becomes metallic when doped with a significant concentration of nitrogen vacancies. We show that the DFT+*U* band structure of SmN represents the physical material over a wide range of nitrogen vacancy doping, and that electrical transport is mediated though in-gap defect states, which project strongly onto majority spin Sm 4*f* states. We have tracked the superconducting and magnetic transitions of SmN and found that the most robust superconductivity is near the breakdown of magnetic order, at the boundary between the FM and AFM phases of SmN and Sm respectively. Our measurements point to the location of a quantum critical point in the SmN-Sm phase diagram and further suggest that the observed superconductivity is unconventional S = 1 type.

## Methods

### Thin film growth

SmN thin films were grown in various ultra-high vacuum chambers with base pressures on the order of 10$^{-9}$ mbar (see reference^32^ for details). A range of substrates have been used (Al$_2$O$_3$, Si and SiO$_2$) selected for ease of electron transport and optical measurements. Sm was evaporated at a flux of $\sim$ 1 $\mathrm {\mathring{A}}$/s in the presence of molecular nitrogen at varying pressures from $1~\times 10^{-6}$ mbar to $4~\times 10^{-4}$ mbar to control V$_N$^64^. As a further level of control the substrate temperature during growth was varied in the range of 300 K to 700 K. X-ray diffraction confirmed all films were rock-salt SmN. Once grown to the chosen thickness ($\sim$ 100 nm) the films were passivated with insulating AlN.

### Electrical transport, optical and X-ray spectroscopy measurements

Van der Pauw electron-transport measurements were conducted in a Quantum Design Physical Properties Measurement System. Optical transmission and reflection measurements were conducted at ambient temperature between energies of 0.01–4 eV in a Bruker Vertex 80v Fourier transform spectrometer. Reflection measurements were referenced using an Al film, and the results then adjusted for the finite reflectivity of Al^65^. The optical measurements were modelled using the software package RefFit^66^, as described in Ref.^60^, with the resulting optical conductivity presented here.

The XPS measurements were performed using a Kratos XSAM 800 spectrometer. An Al source was used to provide monochromated K$_\alpha$ X-rays. During analysis, the operating pressure was typically $8\times 10^{-9}$ mbar or better. To remove the AlN passivation layer, the samples were sputtered using Ar$^+$ ions.

### Density functional theory calculations

Density functional theory based calculations were undertaken using Quantum Espresso^67,68^ and recently developed rare earth pseudo-potentials^69^. Self-consistent calculations on the primitive cell were completed using a *k*-mesh with $10\times 10\times 10$ divisions, while super-cell calculations were on a $4\times 4\times 4$ division *k*-mesh. The wave function and charge density cut-off energies were 50 Ry and 200 Ry respectively for all calculations. Following our DFT calculations the output from Quantum Espresso was used to generate maximally localised Wannier functions using Wannier 90^70–72^. The resulting Wannier functions were then used to calculate the DOS and JDOS on denser *k*-meshes of $25\times 25\times 25$ divisions.

The 4*f* electrons of the *Ln*N series are strongly correlated and thus require careful treatment beyond the traditional DFT methods^35,39^. In the basic DFT (i.e. LSDA) the 4*f* states are found at or near the Fermi energy for most of the stoichiometric *Ln*N. In reality the strongly correlated nature of these electrons pushes the filled states below and unfilled states above the Fermi energy. This physics can be approximated using the DFT+*U* method where the behaviour of the correlated orbitals is determined by an adjustable parameter *U*. In the present study two *U* parameters are used, as described first in reference^35^. One to account for the strongly correlated 4*f* states ($U_f$), and a second applied to the 5*d* states ($U_d$). Selection of the Hubbard parameters is guided by recourse to experimental results, and is discussed in reference^45^ and in section “Band structure".

## Data Availability

The data used during this study are available from the corresponding author upon reasonable request.
